# Development of a microarray platform for FFPET profiling: application to the classification of human tumors

**DOI:** 10.1186/1479-5876-7-65

**Published:** 2009-07-28

**Authors:** Sven Duenwald, Mingjie Zhou, Yanqun Wang, Serguei Lejnine, Amit Kulkarni, Jaime Graves, Ryan Smith, John Castle, George Tokiwa, Bernard Fine, Hongyue Dai, Thomas Fare, Matthew Marton

**Affiliations:** 1 Translational Sciences, Department of Molecular Profiling, Merck Research Laboratories, 401 Terry Ave N, Seattle, WA 98109, USA; 2 Sirna Therapeutics, LLC, Department of Lead Discovery, 1700 Owens Street, San Francisco, CA 94158; 3 Custom Analysis, Department of Molecular Profiling, Merck Research Laboratories, 401 Terry Ave N, Seattle, WA 98109, USA; 4 Custom Analysis Informatics, 33 Avenue Louis Pasteur, Boston, MA 02115; 5 Gene Expression Laboratory, Department of Molecular Profiling, Merck Research Laboratories, 401 Terry Ave N, Seattle, WA 98109, USA; 6 Molecular Informatics, Department of Molecular Profiling, Merck Research Laboratories, 401 Terry Ave N, Seattle, WA 98109, USA; 7 Epigenomics, Inc., 1000 Seneca Street, Seattle, WA 98101; 8 Clinical Molecular Profiling, Department of Molecular Profiling, Merck Research Laboratories, 401 Terry Ave N, Seattle, WA 98109, USA; 9 Genentech, Inc., One DNA way, South San Francisco, CA 94080; 10 Advanced Technology Solutions, Department of Molecular Profiling, Merck Research Laboratories, 401 Terry Ave N, Seattle, WA 98109, USA; 11 External Scientific Affairs, Sumneytown Pike, PO BOX 4, West Point, PA 19486

## Abstract

**Background:**

mRNA profiling has become an important tool for developing and validating prognostic assays predictive of disease treatment response and outcome. Archives of annotated formalin-fixed paraffin-embedded tissues (FFPET) are available as a potential source for retrospective studies. Methods are needed to profile these FFPET samples that are linked to clinical outcomes to generate hypotheses that could lead to classifiers for clinical applications.

**Methods:**

We developed a two-color microarray-based profiling platform by optimizing target amplification, experimental design, quality control, and microarray content and applied it to the profiling of FFPET samples. We profiled a set of 50 fresh frozen (FF) breast cancer samples and assigned class labels according to the signature and method by van 't Veer et al [1] and then profiled 50 matched FFPET samples to test how well the FFPET data predicted the class labels. We also compared the sorting power of classifiers derived from FFPET sample data with classifiers derived from data from matched FF samples.

**Results:**

When a classifier developed with matched FF samples was applied to FFPET data to assign samples to either "good" or "poor" outcome class labels, the classifier was able to assign the FFPET samples to the correct class label with an average error rate = 12% to 16%, respectively, with an Odds Ratio = 36.4 to 60.4, respectively. A classifier derived from FFPET data was able to predict the class label in FFPET samples (leave-one-out cross validation) with an error rate of ~14% (p-value = 3.7 × 10^-7^). When applied to the matched FF samples, the FFPET-derived classifier was able to assign FF samples to the correct class labels with 96% accuracy. The single misclassification was attributed to poor sample quality, as measured by qPCR on total RNA, which emphasizes the need for sample quality control before profiling.

**Conclusion:**

We have optimized a platform for expression analyses and have shown that our profiling platform is able to accurately sort FFPET samples into class labels derived from FF classifiers. Furthermore, using this platform, a classifier derived from FFPET samples can reliably provide the same sorting power as a classifier derived from matched FF samples. We anticipate that these techniques could be used to generate hypotheses from archives of FFPET samples, and thus may lead to prognostic and predictive classifiers that could be used, for example, to segregate patients for clinical trial enrollment or to guide patient treatment.

## Background

While genome-wide mRNA profiling with microarrays has been widely used with fresh frozen (FF) total RNA, few discovery platforms have reliably been applied to formalin-fixed paraffin-embedded tissue (FFPET) samples. Some approaches that assay fewer transcripts (e.g., DASL [Illumina, San Diego, CA] or HTG [Tucson, AZ]) are promising, but do not allow for unbiased discovery of diagnostic signatures, which requires a genome-wide profiling method [2,3]. For example, the DASL assay has been modified to accommodate several thousand genes and was used to derive an expression signature correlated with survival in hepatocellular carcinoma patients [4]. While such sub-genomic platforms may be useful when the target genes are known, applications such as the discovery of biomarkers and the development of *de novo *classifiers specifically benefit from a more comprehensive genomic profile. Standard extraction and amplification microarray protocols (e.g., the Arcturus Paradise Reagent System [5] and NuGEN's Ovation FFPE method [6]) and array platforms (Affymetrix Human X3P arrays [7,8]) have been adapted to handle FFPET samples, but typically generate detectable present call rates only on the order of 30% [7-9], and if the block is more than 5 years old, present call rates can drop below 20% [10]. While successful whole-genome profiling from FFPE has recently been shown with one color Affymetrix arrays [11], similar approaches for two-color arrays have not yet been developed.

The importance of expression-based classification of human tumors to predict treatment response or disease outcome is highlighted by recent publications [9,12]. van't Veer et al [1] were one of the first to apply microarray methods for profiling a group of young, lymph node negative patients with primary invasive breast carcinoma with known treatment outcome. Using the initial profiling data as training and test sets [1,13], a 70-gene prognostic signature was identified to predict disease progression for early-stage estrogen receptor-positive and negative tumor patients. The test was subsequently developed into the first DNA microarray-based expression in vitro diagnostic test (MammaPrint™) cleared by the FDA for actionable decisions in the risk-management of disease. However, these landmark analyses and the subsequent tests have two disadvantages – they require as much as 5 μg total RNA and they require FF tumor samples [14].

In the clinical diagnosis of patients with cancer, it is routine to obtain a FFPET sample, but generally rare to obtain a FF sample. Consequently, the requirement of FF samples has limited expression profiling to patients treated at specialized research centers. The ability to use FFPET samples would make this technology available for virtually all cancer patients both in the context of retrospective analyses of banked samples and clinical trials seeking to identify molecular tumor characteristics associated with patient outcomes to treatment. Being able to do such analyses from FFPET samples would simplify sample biopsy collection requirements and enable retrospective studies to develop and test hypotheses for prognostic classifiers for other cancers. As additional tests are developed and as molecular profiling methods mature, health-care providers will come to rely more on such classifiers in the risk-management of disease.

In this report, our primary focus was to develop sample processing and classification methods with archived FFPET samples for hypothesis generation. To this end, we optimized a microarray platform and applied it to the profiling of FFPET samples. We demonstrated that FFPET samples can be accurately assigned to class labels using a classifier developed from fresh frozen samples, and we show that a classifier derived from FFPET samples that performs well in classifying FF samples can be developed.

## Methods

### Human tissue samples and reagents

Matched pairs of Fresh Frozen and Formalin-Fixed Paraffin-Embedded breast cancer samples were obtained from Genomics Collaborative (Bioserve, Beltsville, MD) and Cytomyx (Lexington, MA). Colon carcinoma tissues were also from Cytomyx. RNA extraction reagents for FFPET samples were obtained from Epicentre Biotechnologies (Madison, WI). Jurkat total RNA and amplification reagents used in this study were from Ambion (Austin, TX). Matched FF and FFPET liver and muscle RNAs were obtained from MPI Research (Mattawan, MI). The Universal Human Reference (UHR) total RNA was obtained from Stratagene (La Jolla, CA). Cy-dye reagents were from GE Health sciences (Piscataway, NJ). Quantitative PCR reagents were purchased from Applied Biosystems (now Life Technologies, Foster City, CA). Microarrays were designed at Rosetta and manufactured by Agilent Technologies (Santa Clara, CA).

### RNA extraction from matched FF and FFPET samples

Total RNA from FF samples was extracted by the vendor (Bioserve, Beltsville, MD) immediately prior to shipment. For FFPET samples, the extraction protocol is adopted from MasterPure RNA Purification kit (Epicentre Biotechnologies, Madison, WI). Briefly, three 10 μm sections were subjected to paraffin solubilization with xylene. Tissue was pelleted from solution by centrifugation and residual xylene was removed by two ethanol rinses. The tissue pellets were then air dried and digested overnight in a lysis buffer with Proteinase K. Digested protein and other cellular components were removed by ammonium acetate precipitation and centrifugation followed by a DNase I treatment of the resulting supernatant. A second ammonium acetate precipitation to remove any residual protein was then performed prior to ethanol precipitation and nuclease-free water rehydration of the purified total RNA.

### Quantitative PCR for Total RNA and cRNA QC

For measuring the relative quality of total RNA from FFPET samples, primer pairs for two house-keeping genes, GAPDH and ribosomal protein L13a, were used to estimate the integrity of the total RNA (Table 1). For measuring the relative abundance of transcripts in the amplified cRNA, the primer pair for gene amyloid beta precursor protein binding protein 1 (APPBP1) was used. Total RNA or cRNA (100 ng or 50 ng per reaction, respectively) from FF Jurkat were used as a control for the corresponding qPCR experiments. Besides the specific primers, all reagents for qPCR assay were obtained from Invitrogen (Carlsbad, CA). The qPCR experiments were conducted with the ABI 7900HT according to the recommended protocol for the SYBR green detection. The experimental condition for each primer pair is optimized with good quality FF samples before applying to FFPET samples. Following the reverse transcription reaction, each cDNA sample was split into 4 wells for performing PCR reaction and measurement. The qPCR result was processed with the associated SDS software and the data was reported as raw Ct for each sample.

**Table 1:** List of qPCR primers

Gene Name	Primer Description	Sequence
Ribosomal protein L13a	RPL13a Forward	CACTTGGGGACAGCATGAG
	
	RPL13a Reverse	GTAACCCCTTGGTTGTGCAT

Glyceraldehyde-3 Phosphate Dehydrogenase	GAPDH Forward	AGTCCCTGCCACACTCAGTC
	
	GAPDH Reverse	CTGTGAGGAGGGGAGATTCA

Amyloid beta precursor protein binding protein 1	APPBP1 Forward	TCTTCGAGTGGTAAGATGTCGATCC
	
	APPBP1 Reverse	ACCCGAAGGCAATTACAGTTTCAAT

### RNA target preparation (cRNA amplification)

The RNA target was amplified by modifying an industry-standard amplification protocol (Ambion, Austin, TX). The original protocol employs oligo-dT priming for first strand cDNA synthesis, in which an oligo-dT primer incorporates a T7 RNA polymerase promoter, necessary for subsequent in vitro transcription of RNA (Supplementary Figure 1). Besides the modification of the procedure for a semi-automated operation, the initial total RNA input for the first round amplification was optimized at 100 ng for both fresh frozen and FFPET RNA samples; the input of the second round amplification was normalized to 500 ng of the first round cRNA product per reaction. We found it necessary to keep reaction conditions for the second round amplification identical for all samples: 500 ng of each cRNA product from the first round was dried down and resuspended in a uniform volume. To ensure the effective synthesis of the second strand cDNA synthesis in the second round of amplification, RNase H was added into the reaction mixture to degrade cRNA in the RNA-DNA hybrid before the addition of the oligo-dT T7 RNA polymerase primer. The cDNA and cRNA purification were performed with the Agencourt RNAClean system (Agencourt, Beverly, MA,) and RNeasy (Qiagen, Valencia, CA) methods. The cRNA and cDNA concentration were measured by the standard UV absorption method with a microplate reader (SpectraMax 190, Molecular Devices, Sunnyvale, California).

**Figure 1:**
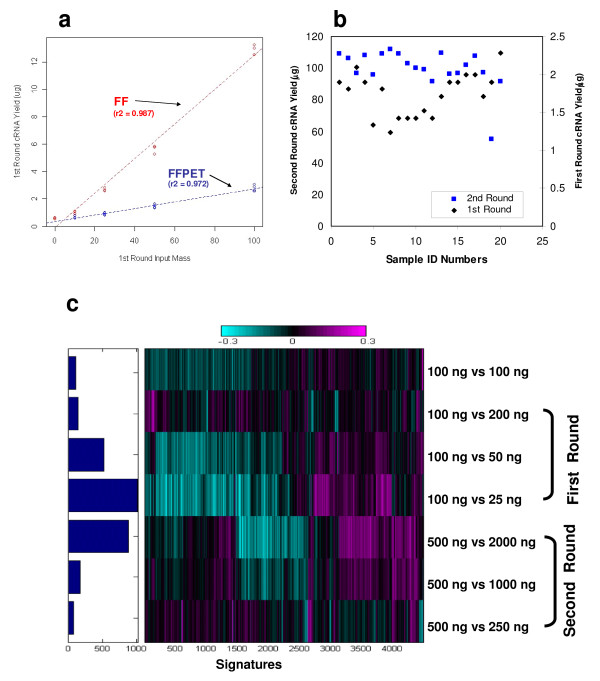
**a) Dose response curve showing cRNA yield from the first round RT-IVT amplification as a function of total RNA input for Jurkat total RNA sample from FF or for a colon carcinoma RNA derived from FFPET**. b) Plot showing the distribution of cRNA yield from the first and second round RT-IVT amplifications from a representative set of FFPET samples. First round and second round yields are shown as black diamonds and blue squares, respectively. The first round RT input is 100 ng and second round RT input is 500 ng. c) One-dimensional heatmap of hybridization experiments starting with the same total RNA but with different mass input, showing that both the first and second round can tolerate more than 2-fold of sample mass imbalance. The left panel bar graph shows the number of significant signatures, and the right panel heatmap shows total number of genes selected for these analysis.

### Target cRNA labelling and hybridization

Five micrograms of cRNA from each experimental sample were dried down and labelled with Cy3 dyes (Invitrogen), and co-purified with the same mass of cRNA from the UHR pool labelled with Cy5. Hybridizations were done in fluor-reversed pairs as described [15]. Labeling, microarray hybridizations, scanning of the slides, and image processing were described in Marton et al [15].

### Re-ratio method

Since we are mostly interested in ratio profiles between FFPET samples, we developed methods to recover such information by re-ratioing the ratio profiles derived from each array experiment between the FFPET samples and the corresponding hybridizations with UHR as the reference. Re-ratio of the ratio profiles effectively cancels out the UHR profile while accounting for dye labelling biases and leaving only the FFPET profiles of interest. In other words, given a typical configuration in which the common reference (*C*) exists in two array-based ratio profiles, if hybridization 1 consists of the ratio *C *versus *A *(*A/C*), and hybridization 2 consists of the ratio *C *versus *B *(*B/C*), re-ratioing of *A/C *and *B/C *creates a new ratio experiment *B *versus *A *(*A/B*).

### Clustering, statistical analysis and classification methods

Gene clustering was performed independently by using an agglomerative hierarchical algorithm [16]. Pairwise similarity metrics among genes are calculated on the basis of expression ratio measurements across all experimental samples. A detailed procedure and calculation was described in van 't Veer et al [1]. We defined the Odds Ratio (OR) for this study as the ratio of odds of developing distant metastases within 5 years for a patient in the referenced study with a tumor characterized by a poor prognosis signature to the odds of developing metastases without this signature (2 × 2 Table). The development of the FFPET-based classifier using supervised classification was performed with following three steps: 1) selection of discriminating candidate genes by their correlation with the category; 2) determination of the optimal set of reporter genes using a leaving-one-out cross validation procedure; 3) prediction of class label (or prognosis or diagnostic) based on the gene expression of the optimal set of reporter genes. The significance of the classifier was estimated by the OR and the 95% confidence interval. The *p*-value associated with the OR was calculated by Fisher's exact test. More detailed of statistical analysis and classification were reported by van 't Veer et al [1].

### Novel DNA microarray probe design

Human 44 k v1.1 array  and the HumFFPET 44 k v2.0 array were the default arrays for profiling FF samples and FFPET samples, respectively. The design of the HumFFPET 44 k v2.0 array involved a development array, HumFFPET 44 k v1.0, which itself was based on the Human 44 k v1.1 array. The development array (HumFFPET 44 k v1.0) was initially designed to evaluate several oligonucleotide selection criteria rule sets (see Table 2) and 3' distance as potential factors for modifying the probe design to improve FFPET profiling. The final oligonucleotide selection criteria parameters for the HumFFPET 44 k v2.0 array were derived from hybridization data on the HumFFPET 44 k v1.0 development array. The unique oligonucleotide selection criteria for the HumFFPET 44 k v2.0 array include modifications to the oligonucleotide set design (OSD) for probe 3' distance, base composition, and increased leniency with the cross hybridization filter.

**Table 2:** Probe content for FFPET 44 k v1.0 development array

**OSD Name**	**# of Probes**	**OSD Parameter**
OSD 0	3,996	Current default OSD
OSD 1	3,938	Current default OSD with increased weighting towards 3' end
OSD 2	4,003	Current default OSD weighted towards 100 nt from 3' end
OSD 3*	29,931	Tile at 30 nt up to 400 nt from 3' end
OSD 4	4,003	Tiled house keeping probes to 1000 nt from 3' end

Array represents approximately 4,000 unique genes with probes for OSD0 – OSD3. *OSD 3 was tiled, but any probes obviously failing standard oligonucleotide selection criteria for repeat sequences, bad base composition, and cross-hybridization were removed.

For each probe, the potential to cross-hybridize was calculated by comparing the probe sequence against the human transcriptome using sequence similarity and the 'BLAST' algorithm. The binding affinities between the probe and the transcript sequences identified by BLAST were computed in terms of dG (delta G). The cross-hybridization score reflects the smallest difference between the dG value of self binding and the largest dG value of the probe binding to any other molecular species. However, despite making the cross hybridization filters more lenient, we noticed that the cross hybridization can only be minimized since certain probes still contain relatively higher GC content compared to the majority of probes on the array in order to meet the 3' distance requirement. Selection of the genes for the HumFFPET 44 k v2.0 array was kept as close as possible to those on the current default Human 44 k v1.1 array. The human 44 k v1.1 and HumFFPET v2.0 array share 20,327 probes in common while the HumFFPET v2.0 array has 19,231 unique probes that are specifically designed for FFPET samples. Documentation for the HumFFPET 44 k v2.0 array will be available in the Gene Expression Omnibus (GEO) website in support of this publication, and the HumFFPET 44 k v2.0 array pattern will be publicly available through the Agilent eArray ordering system.

## Results and Discussion

Formalin-preserved samples present multiple challenges to whole-genome RNA profiling methodologies. To overcome the extensive degradation, contaminants from the formalin treatment and limited RNA mass availability, we sought to improve 1) robustness of the amplification protocol, 2) quality control assessment of FFPET samples, 3) microarray performance through probe selection and 4) experimental design by validating the use of UHR as the reference channel in two-color hybridization experiments.

### Improvement of the robustness of the RT-IVT amplification protocol

#### Linear dose-response

We started with a commercially available two-round RT-IVT protocol for the target cRNA preparation for non-FFPET samples (Additional file 1) but observed it was not sufficiently robust in our hands to be adopted as a high-throughput, automated protocol. We reasoned it was essential that the protocol have properties such as a linear input-dose response curve and be robust to mass imbalances and that these properties may be dependent on sample mass input. We determined the optimal mass input amounts in both the first and second round RT reactions that provided both a linear dose-response and insensitivity to mass input imbalances. The relationship between the input amount and yield for both FF (Jurkat cell line) and FFPET samples (colon carcinoma) is shown in Figure 1a. The cRNA yields for FF and FFPET are directly proportional to the corresponding total RNA input with a good linear dose-dependence curve; we note that the cRNA yield from the FF total RNA is significantly higher (Figure 1a). It is our experience that a linear relationship between total RNA input and cRNA output is necessary to ensure representative amplification and to avoid artifacts or false positive gene expression signatures on different microarray platforms. Figure 1b shows the cRNA yield distribution of a set of 20 breast carcinoma FFPET sample. The input for the first round amplification was 100 ng total RNA, which yielded 1–2 μg cRNA; the input for the second round was 500 ng, which yielded 60–115 μg cRNA. In the first round amplification, the input total RNA only contains a small fraction of amplifiable RNA (mRNA), usually less than 1% for FFPET samples. However, the input of 500 ng cRNA for the second round is derived from oligo dT-based amplification from the first round, and all cRNA molecules should contain the specific polyT tails for further amplification. Essentially, all cRNA molecules with polyT tails in the second round are expected to be amplifiable following the conversion into T7-promoter-containing double strand cDNA. Thus, we attribute the lower yields in the first round to the lower amount of amplifiable mRNA relative to the second round amplification. This is consistent with the prediction that cRNA input used in the second round contains a greater percentage of amplifiable RNA than the total RNA used in the first round amplification.

#### Robustness to sample mass imbalances

The rationale for using Jurkat RNA is to model the tolerance of the platform to sample mass variations in terms of mRNA content when the expression data of experimental samples are presented as ratios. Each microarray platform has different degrees of tolerance to sample mass imbalance, which can result from either operational variations during the sample preparation process, or from the variation of amplifiable mRNA content in the samples. For example, different FFPET samples contain very different amounts of amplifiable mRNA even when measured total RNA concentrations are the same (data not shown). We used a mass imbalance of intact Jurkat RNA to assess the potential impact of degradation and chemical modifications that would vary across FFPET samples. While evaluating imbalanced FFPET samples directly was more appealing, we reasoned that the primary impact from the degradation and chemical modifications of the fixation and embedding procedures would be loss of amplifiable mRNA, which the Jurkat RNA experiment adequately approximates.

To quantify the degree to which the amplification steps are susceptible to mass imbalance, we performed titrations of input mass for the first round amplification with 25 to 200 ng of Jurkat total RNA sample to model the impact of mass imbalance on microarrray data quality. Following the first round amplification using different mass inputs, a fixed amount (500 ng) of cRNA derived from each input mass titration in the first round amplification was used for the second round amplification. The resulting cRNA from the second round amplification was labelled and hybridized in fluor-reversed pair that was formed to reflect the initial first round mass imbalance between the reference input of 100 ng and the other mass inputs which were originally titrated in the first round amplification. The same experiments were done to titrate input mass for the second round amplification by holding the mass of the first round constant at 100 ng and varying the second round from 250 to 2,000 ng, with the input of 500 ng used as the baseline to form the reference for the different fluor-reversed pairs.

In these mass imbalance experiments, hybridizations of fluor-reversed pairs that are formed between different mass input are still defined as 'same-vs-same' hybridizations since the exactly same mRNA-containing total RNA are used in the amplification whether the input varied in the first or the second round. If there is no amplification bias resulting from the initial mass inputs, either for the first round or the second round, then these same-vs-same hybridizations should have shown no signatures of differential expression beyond background level. Same-vs-same hybridization data are presented as a heat map in Figure 1c, which shows the second round amplification is less susceptible to spurious signatures resulting from mass imbalance than the first round. Fewer than 500 differential signature genes were detected in the hybridization with up to 2-fold mass imbalance for the first round titration experiment and below 200 for the second round, which corresponds to ~1% and 0.5%, respectively, of false positive rate for the microarray hybridization with 44,000 gene probes. These false positive rates are within the background level normally predicted and defined as the pass and fail metrics for different microarray platforms (Affymetrix and Agilent). Based on the mass imbalance experiment and our operational requirement of the high-throughput amplification procedures, the optimal sample mass inputs for the first and second round amplification were determined to be 100 and 500 ng respectively. The selected inputs have a high tolerance to unintended mass imbalances either up or down from the intended input, which serves as a buffer against the potential sample quality variation inherent in FFPET sample sets and the operational variation during the amplification process.

### Use of quantitative PCR for assessing the relative quality of FFPET total RNA and amplified cRNA

The extent and nature of RNA degradation in FFPET blocks depends on FFPET preparation method, length of storage and storage conditions [9,17,18]. While the RNA extraction procedure can be optimized to increase RNA yield (data not shown), most FFPET blocks were not prepared with RNA quality preservation in mind. Even if sufficient amounts of total RNA can be recovered from poorly prepared and stored FFPET blocks, one cannot be certain the microarray experimental results derived therefrom will yield biologically meaningful data, even after applying bioinformatic and data processing approaches to compensate for the effects of degradation. For FF samples, the quality the total RNA can be assessed by the integrity of the 18S to 28S ribosomal RNAs (measured by abundance ratio) or the RNA Integrity Number (RIN) [19]. It is not feasible to employ similar methods for assessing the quality of FFPET-derived total RNA samples. Thus, there is a need to measure the relative total RNA quality of FFPET samples and to determine whether data are impacted by poor total RNA quality or by the procedures used to store or process the samples. In particular, we sought to determine whether the quality of the microarray hybridization data for a given sample could be predicted from the integrity of the starting total RNA or the amplified cRNA prior to microarray hybridization.

First, we noted that mean log ratio of some samples displayed a dependence on the distance of the probe to the 3' end of the message. We reasoned that a non-zero slope of this plot indicates a bias in the data quality (i.e., a data artifact), and that the 3' slope of mean log ratio could be used as a key quality metric for microarray hybridization. In fact, the 3' slope metric is analogous to the RNA Degradation metric [20] on Affymetrix microarrays, which was originally developed to measure bias in array data due to degradation of total RNA. In an ideal experiment, number of signatures should not correlate to 3' slope. From a plot of the slope vs Ct, we determined the region of no correlation to Ct is around a slope of 0.15; therefore, we use 0.15 as the threshold for 3' bias. We suspect the 3' slope metric is a measure of variation in cRNA length introduced by amplification bias or RNA quality. The variation in cRNA length is likely to be induced during first strand cDNA synthesis and likely can be applied to any mRNA amplification method utilizing reverse transcription.

**Figure 2:**
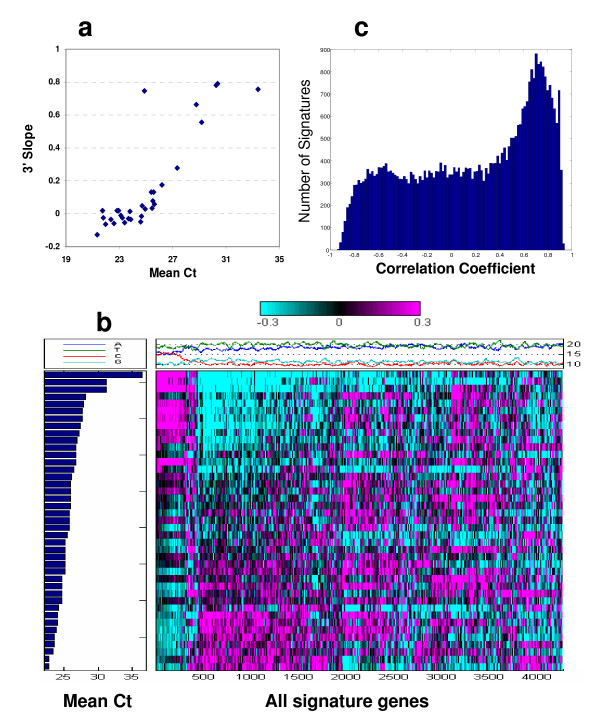
**a) The mean Ct of the housekeeping gene (GAPDH) is correlated with the 3' slope of mean log ratio of the signatures from FFPET muscle issues**. The 3' slope of mean log ratio is one of the key quality metrics for microarray hybridization, which is the mean expression ratio of all genes as a function of distance to the 3' end of the transcript. Good quality samples that show no amplification bias will have a 3' slope of zero. b) One-dimensional heatmap of FFPET hybridization experiments starting with cRNAs of different qualities sorted by Ct count of the APPBP1. The Ct count for gene APPBP1 is a measure of the relative abundance of 'specific transcript' (amplifiable RNA) in the amplified cRNA for each sample. A distinctive gene expression pattern is correlated with the Ct count of cRNA for each individual hybridization. At *p*-value of 0.05, 4,500 genes are selected in this plot. The top panel shows the relative GC content for the corresponding signatures. c) Histogram showing the number of genes in each correlation between the mean Ct count of the amplified cRNA and the detected expression pattern. In an ideal hybridization, there should be no correlation between the Ct count of the APPBP1 for the amplified cRNA and the detected expression pattern, either positive or negative. However, the histogram of correlation coefficient with Ct counts for all genes detected on the FFPET array indicates that a significant fraction of genes is correlated or anti-correlated with the Ct count of the APPBP1 for the amplified cRNA, suggesting that the sample quality rather than the biology of FFPET samples dictates the correlation.

Then, we developed a qPCR-based assay to measure the relative abundance of transcripts in the total RNA and the amplified cRNA to quantify the relative quality of FFPET-derived total RNA as a way to relate the RNA quality to the subsequent microarray hybridization quality. Then, as we had done with FF samples, we started by selecting a set of FFPET samples that had been previously profiled and that cover a range of microarray data quality, and then assayed several housekeeping transcripts by qPCR. Figure 2a shows the correlation of abundance (mean Ct) of a housekeeping gene, GAPDH, in total RNA versus the 3' slope of mean log ratio of all genes from FFPET breast cancer tissues. To measure the relative abundance of transcripts in the amplified cRNA, we selected a set of cRNAs that have been previously profiled with known data. The strong correlation of signatures to the Ct counts of amyloid beta precursor protein binding protein (APPBP1) indicates bias in cRNA specificity. The higher is the Ct counts the lower is the specificity. It is difficult to determine a sensitive universal threshold because abundance of transcripts depends on the tissue examined. Nevertheless, we established that samples with APPBP1 Ct > 29 should not be used in downstream analysis because majority of cRNA is likely to be non-specific material. As shown in Figure 2b, the relative abundance (mean Ct) of mRNA for APPBP1 is correlated to the expression pattern of the selected samples. We noticed that there is a large fraction of signatures either positively or negatively correlated with Ct count of the corresponding cRNA (Figure 2b). It is also important to note that these signatures are also enriched for the C content of the array probe as observed in the upper portion of Figure 2b. The lack of specificity of these C-rich probes reinforces the conclusion that these signatures are spurious regulations. Furthermore, the skewed distribution of ratio correlation of signature genes with Ct counts, along with the enrichment for C content in the correlated array probes, indicates that the sample quality rather than biology caused the skewed distribution of ratio correlation (Figure 2c).

In summary, the hybridization data of a sample of ideal quality should show no correlation between the Ct count of the total RNA or the amplified cRNA and the detected expression pattern, either positive or negative. Since the measured Ct count from the total RNA and amplified cRNA correlate with the quality of microarray hybridization (Figure 2), the qPCR method can be utilized as a cost- and time-effective manner to assess the relative quality of total RNA and cRNA for FFPET microarray assays. It should be noted that measured Ct count in terms of sample quality will be contextual not only on the basal expression of the transcripts in the sample type of interest, but also within the experimental FFPET sample set. Our approach of estimating FFPET sample quality using a qPCR method and the method's effectiveness at predicting array performance (at either total RNA and cRNA levels) improves our confidence in the data quality of the FFPET profile.

### Development and optimization of an FFPET array

**Figure 3:**
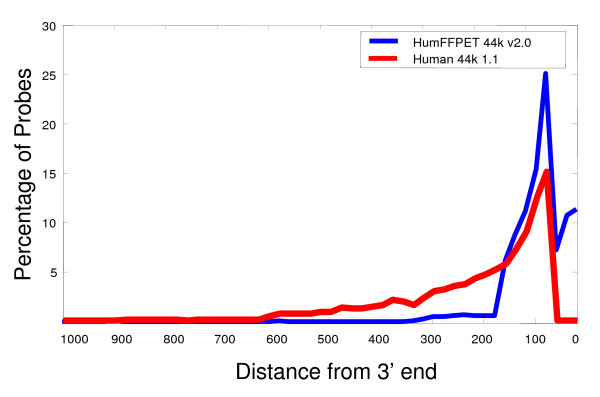
**Percentage of probes as a function of distance from the 3' end of a transcript to the 3' end of the probe for the HumFFPET 44 k 2.0 and the standard Human 44 k v1.1 arrays**. Note that over 95% of probes on HumFFPET v2.0 are within 200 bases to each transcript 3' end.

Standard microarrays with probes approximately 500 nucleotides or greater from the 3' end have been found to be ill-suited for FFPET profiling. Therefore, we designed an array with probe content more suited to nature of FFPET samples and less susceptible to cross-hybridization by optimizing probe distance to the 3' end of the transcript, the probe base composition and the OSD cross-hybridization filter. While we had preliminary success at obtaining gene expression data from FFPET samples on a 44 K array (Human 44 K) designed for FF samples, the raw intensity of signals measured on the array drops off more precipitously for FFPET samples than FF samples as the probes' distance from the 3' end is increased. This is due to the extensive degradation of total RNA and the use of 3' poly-A priming amplification approach. One way to overcome the reduced intensity of hybridizations with cRNA from the 3' poly-A priming method is to optimize the probe content and composition on the array to reflect the proximity of amplified cRNA sequence of each transcript to its 3' end from degraded FFPET samples. To this end, we designed an array specifically suited to profiling FFPET samples with probe sequences within the first 400 nucleotides of the 3' end of each transcript (Figure 3).

**Figure 4:**
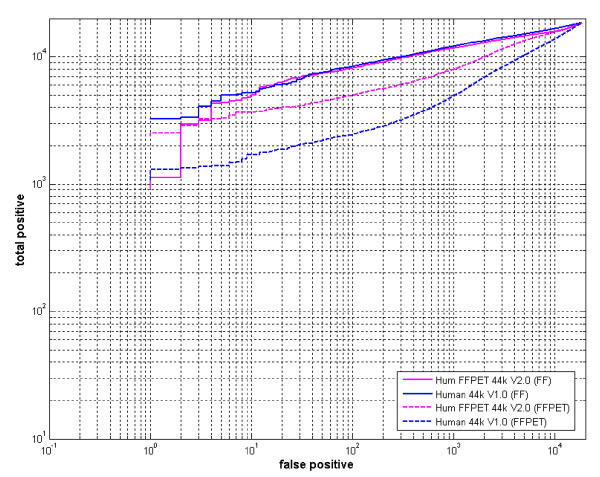
**ROC curve analysis showing sensitivity and specificity of Hum FFPET 44 k and Human 44 k microarrays with cRNA generated from FF and FFPET samples**. The ROC curves demonstrated the sensitivity and specificity of microarray hybridizations with cRNAs from FF samples on Hum FFPET 44 k v2.0 (solid red line) and Human 44 k v1.0 (solid blue line) and cRNAs from FFPET samples on Hum FFPET 44 k v2.0 (dashed red line) and Human 44 k v1.0 (dashed blue line). The number of false positives on the x-axis indicated the specificity (data derived from liver versus liver arrays); the number of positive on the y-axis indicated the sensitivity (data derived from liver versus muscle experiments). Each ROC curve was generated with data from one pair of liver versus liver arrays against data from one pair of liver versus liver arrays, and one representative ROC curve of three plots was shown for each method.

To evaluate the performance of the new FFPET array (HumFFPET 44 k 2.0), a set of matched FF and FFPET liver and muscle samples were used to measure the sensitivity and specificity of the HumFFPET 44 k 2.0 array in comparison with our standard Human 44 k array (see methods for details). To compare the overall performance of the FFPET and standard array designs, we used a ROC curve analysis [21] to show that the FF samples perform almost identically on either array; on the other hand, the FFPET samples perform significantly better on the HumFFPET 44 k 2.0 array than on the Human 44 k array in terms of sensitivity and specificity (Figure 4). These data indicate that the HumFFPET 44 k 2.0 array could significantly improve FFPET profiling with a minimal impact on FF RNA profiling.

Using the HumFFPET 44 k 2.0 array with a set of FF and FFPET samples, we found that the measured expression ratio correlation between FF and FFPET samples is also improved on the HumFFPET 44 k 2.0 array (r = 0.80) over the Human 44 k array (r = 0.70, Additional file 2), which compared favorably to correlation observed by others [11]. While the performance of FFPET RNA profiling would not be expected to match that from FF samples, the correlation between matched FFPET and FF samples can be used as a way to optimize an FFPET profiling platform (including microarray and quantitative PCR). Our results suggest that the optimization of probe design on HumFFPET 44 k 2.0 array could improve overall performance of the platform for the profiling of FFPET samples.

### Determination of the Optimal Reference Sample for FFPET Profiling

An experiment on the Agilent two-color microarray platform requires the pairing of differentially-labelled experimental and reference samples. Frequently, samples are hybridized to two separate slides in fluor-reversed pairs. The competitive hybridization of differentially labelled samples in the two-color array platform has the potential for increased consistency and reduced susceptibility to protocol variation [15]. The fluor-reversed hybridization is performed to minimize dye bias when two samples are labelled with different dyes. Initially, we focused on self-reference pools because previous studies [21] clearly showed that for optimal microarray performance, the reference pool should be as similar to the experimental samples as possible (i.e., a self-reference pool). However, such self-reference pools present several challenges, including that they require twice as much sample and that poor quality samples in the pool can skew the expression data and create data artifacts.

**Figure 5:**
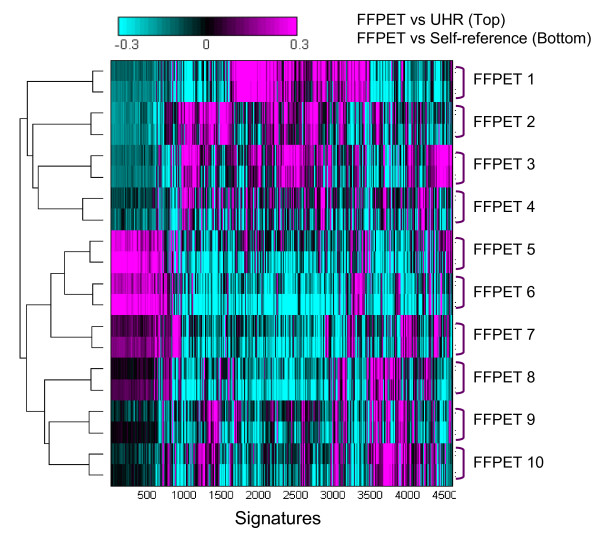
**Two-dimensional hierarchical clustering of self-reference pool vs experiments and the universal reference pool vs experiments**. Identical set of cRNA from FFPET samples are hybridized in pairs with the self-reference pool of FFPET samples and the universal human reference samples. All hybridizations are co-clustered according to the identity of each individual FFPET sample, regardless of references used in the two-color hybridizations.

To obviate the need to generate a population-specific reference pool, we evaluated the microarray data performance of a commercially available and well-defined RNA sample (the Universal Human Reference; UHR) versus a FFPET-based self-reference pool. UHR RNA is composed of total RNA from 10 human cell lines and has been used widely as a reference in gene-profiling experiments, microarray platform evaluations, and cross-platform comparisons [22]. Using UHR as a reference allows for a controlled pool that can be leveraged across large studies while halving the mass requirements for samples which would have made up a self-reference pool. To effectively compare UHR with a self-reference pool control, we hybridized labelled cRNAs from FFPET breast tumors as the experimental channel either against UHR or against a 10-member self-reference FFPET pool as the reference channel. Results were subjected to two-dimensional clustering, and as shown in Figure 5, all ten samples paired with the UHR reference co-clustered with the experiments using the self-reference pool as the reference channel. With the re-ratio method, it is possible to exclude the effect of UHR and compare the FFPET samples of interest in the corresponding hybridizations with UHR as a common reference. Re-ratio of the ratio profiles effectively cancels out the effect of UHR profile, and leaving only the FFPET profiles of interest. Thus, the data indicate that one can generate the same sorting results when using UHR as when using a self-reference pool. Although differentially degraded samples can adversely impact pool performance, in this case, all 10 members of self-reference FFPET pool were assessed with the developed qPCR method and additionally were known to be good quality FFPET samples from previous microarray hybridizations. In general practice, it would be difficult to know the FFPET sample quality without the qPCR step. Overall, we decided to proceed with UHR as the reference because it provided several logistic advantages and did not have a negative impact on the final microarray results.

**Figure 6:**
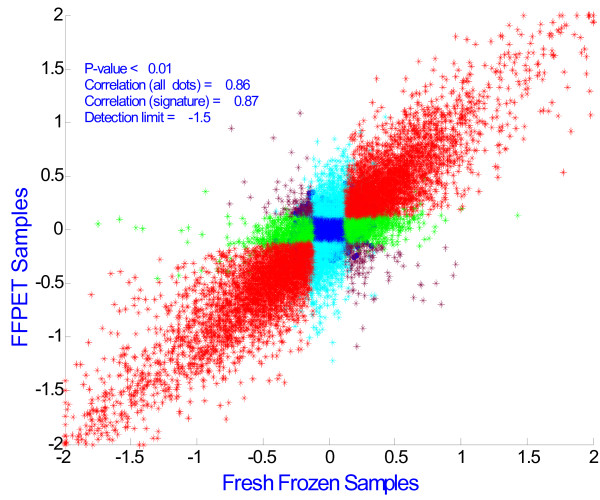
**Correlation of Differential Expression of Muscle and Liver Cancer tissues Between FFPET and Matched FF Samples**. Genes correlated (signatures, either up- or down-regulated relative to reference) are in red; genes detected as signatures in FF but not FFPET samples are green; genes detected as signatures in FFPET but not FF are light blue; genes shown anti-correlation between FF and FFPET samples are brown; genes unchanged are in dark blue.

Before applying the two-color microarray platform for classification, we validated the optimized platform measuring the correlation of gene expression profiles from the matched fresh frozen and FFPET tumor samples. As shown in Figure 6, there is an excellent correlation of measured differential expression ratio of breast and colon cancers between fresh frozen and matched FFPET tumor samples.

### Is FFPET RNA Profiling of Sufficient Quality to Classify Samples?

A number of papers have recently been published utilizing microarray technology to identify prognostic and diagnostic biomarkers as a tool to predict treatment response or disease outcome [12,23]. Such patient segmentation efforts have been successful using microarray data of FF samples. Ideally, one would design a clinical trial to address a specific hypothesis, then collect samples and analyze outcome data for tumor signatures, with validation performed in an independent cohort. While prognostic classifiers of FFPET samples have been reported [4,10,11,23,24], our aim was to determine whether the improvements we describe enabled the generation of hypotheses and classifiers that could be used in subsequent clinical trials. Therefore, we addressed two questions: 1) how will data from FFPET samples perform in predicting class labels derived from FF samples; and 2) how does the predictive power of a FFPET-derived classifier compare to that of a classifier derived from FF samples?

#### Performance of FFPET samples in FF-derived classifiers

To answer the first question, we chose a well established biomarker, the breast cancer prognostic signature identified by van 't Veer et al. [1]. In the van 't Veer's publication, the authors developed a "good prognosis" template using the average expression of the 70 prognostic genes of patients with a good outcome, and then calculated a correlation coefficient to each patient's expression profile to derive a prognostic score. For the present study, we obtained 50 matched pairs (FF and FFPET) of breast cancer samples and calculated the prognostic scores using the methods and templates (both "good template" and "poor template") of van 't Veer et al [1]. Class labels ("good prognosis" and "poor prognosis") were assigned to each of the 50 patients using the FF samples; we then tested how well the FFPET data could match the FF sample class labels (using the same method and templates). Since no clinical or outcome data were available for these samples, the present study does not allow for a direct validation of the prognostic signatures (that is, this was not an objective for this study).

**Figure 7:**
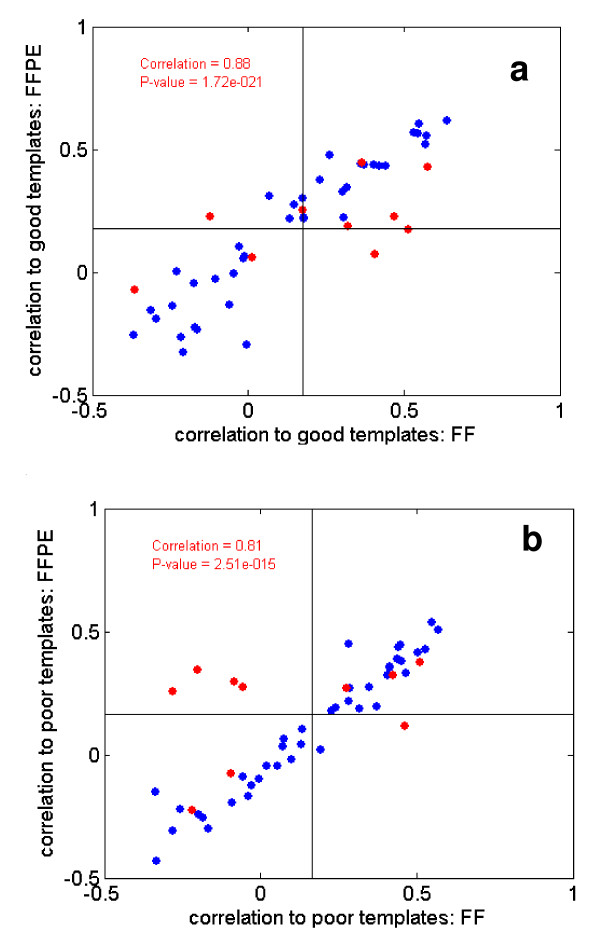
**Correlation plots of prognosis scores for FF and FFPET samples**. Panel A shows the correlation of prognosis scores for FF and FFPET samples using a good prognosis template; panel B shows the same data set using a poor prognosis template. In each case, the correlation is high, despite including samples that were observed to have cross-hybridization artifacts due to poor sample quality (red dots).

Using the prognostic scores from 50 FF samples, we assigned 25 "good prognosis" patients and 25 "poor prognosis" patients according to their signature patterns. Figure 7 compares the prognostic scores from FFPET vs. the scores from the FF samples for good and poor templates separately. As one can see, the scores correlated quite well (r = 0.88 and r = 0.81 for the good and poor prognosis templates, respectively). Except for a few samples which were flagged with poor RNA quality by the qPCR method (Figure 7, red circles), a clear correlation of scores between FFPET and FF samples is observed (Figure 7). When these samples are removed from the analysis, the correlations improve further. When predicting the class labels assigned by the FF samples, the FFPET samples correlate accurately except a few poor quality or boundary samples (for the good template, OR = 36.4, 95% CI: 6.6–201.7, P-value of Fisher's Exact Test: 1.6E-6, average error rate = 16%; with the poor template, OR = 60.4, 95%CI: 10.0–364.4, P = 7.1E-8, and error rate = 12%). Similar to the correlation, the error rate could be attributed to poor quality samples flagged by qPCR. Thus, when the prognosis scores were calculated based on the classifier developed previously from patients who have shown good and poor treatment labels, a clear correlation of scores between FFPET and FF samples is observed (Figure 7).

#### Performance of classifiers derived from FFPET samples

**Figure 8:**
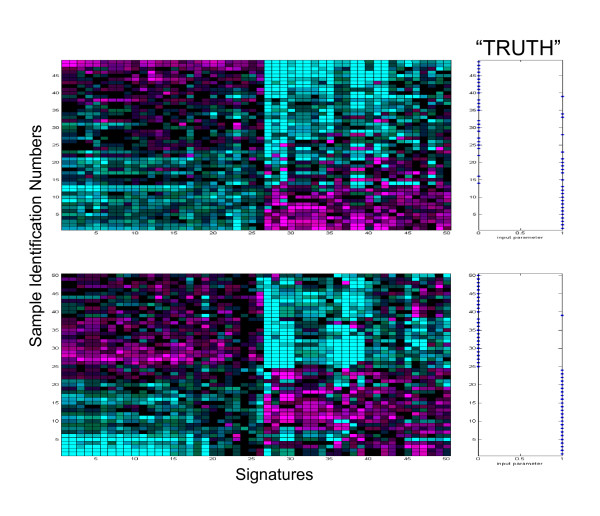
**Two-dimensional hierarchical heatmaps showing classifier discovered from FFPET samples accurately predicts class labels in agreement with FF**. Top panel: samples in FFPET are ranked by classifier score. Leave-one-out cross validation error rate is ~14% (*p*-value of 3.7 × 10^-7^). Bottom panel: FF samples are clustered based on the "prognostic" genes derived from FFPET. The clustering analysis can group all samples correctly except one. The right panel indicates the relative truth as shown in Figure 7.

To determine whether FFPET data is of sufficient quality to discover a classifier, we used the samples divided into "good" and "poor" classes by FF prognosis scores as training sets, and then developed and optimized a classifier using the FFPET gene expression data by a leave-one-out cross validation (LOOCV, including re-selecting features) process to select "prognostic" genes based only on FFPET profiling data. The FFPET-derived classifier prediction accuracy was then evaluated against the FF samples. As shown in Figure 8 (top panel), the classifier has a LOOCV error rate of ~14% (p-value = 3.7 × 10^-7^), with errors mostly coming from poor quality samples flagged by qPCR. If poor quality samples as indicated by qPCR are excluded, the error rate will be 10% or less, mostly caused by samples originally very close to the threshold. More importantly, the classifier derived from the FFPET samples is confirmed by the FF sample data (Figure 8 bottom panel). The derived classifier, when applied to FF data, can predict all the FF sample flags except one, providing strong evidence that classifiers developed from FFPET samples are functional.

#### Comments on the use of FFPET samples for personalized medicine

Enabling personalized medicine in the near future will rely to a large extent on extracting data from well-annotated archived samples for which the three-to-five year outcome of the subject is known. We consider this approach as a bridging strategy for patient stratification and enrollment, during which time the hypotheses are tested and confirmed. Part of this strategy includes integrating FDA guidelines relating to retrospective sample and data mining; key factors in this regard are (1) storage conditions; (2) samples are representative of intended use; (3) samples meet inclusion/exclusion criteria; and (4) performance is comparable to that expected from a prospective study [25]. The FDA has expressed reluctance to accept retrospective data as support for a label that will be used to make clinical decisions because the protocol was not an integral part of development and may not meet the agency's scientific standards for the assay [26]. Therefore, while we demonstrated the ability to develop hypothesis-generating classifiers from FFPET samples based on concordance with classifiers from FF samples, we anticipate significant work remains to validate the results.

## Conclusion

We demonstrated the ability to derive gene expression-based classifiers from FFPET data that sort patient samples into class labels that recapitulates the sorting of FF samples. The method involved the development of an optimized microarray platform with two-round RT-IVT amplification using 100 ng of total RNA input for the target preparation, a quantitative PCR method for assessing the relative quality of FFPET samples and a custom microarray with content and probe features specifically optimized for FFPET profiling. We found this microarray platform reliably and reproducibly measured differential gene expression of FFPET samples with a good correlation to corresponding FF samples. FFPET samples were correctly assigned to class labels developed from FF-derived classifiers. Although we cannot directly attribute the success of the classifiers to the optimizations performed on the profiling platform, further study with FFPET samples also showed our platform is of sufficient quality to enable hypothesis generation that could be validated with FF or FFPET samples in controlled, well-designed clinical trials.

## Competing interests

The authors declare that they have no competing interests.

## Authors' contributions

SD, RS, JG carried RNA extraction optimization, amplification, and hybridization. SD, SL, HD, TF, GT, BF and MM contributed to the design of the studies. MZ and MM drafted the manuscript. AK and JC designed the FFPET array. HD and YW developed the classifier and led the interpretation of the data. All authors read and approved the final manuscript.

## Supplementary Material

Additional file 1 **Two-round amplification workflow**. Diagram of the Two-round amplification work flow used for amplification of total RNA Click here for file

Additional file 2 **FF to FFPET correlation**. The correlation between the FF and FFPET samples is increased significantly on the HumFFPET 44 k array 2.0 when compared to the Human 44 k v1.1 array. Click here for file
