# Supplementary figures and images for: Development of a microarray platform for FFPET profiling: application to the classification of human tumors
Source: J Transl Med. 2009 Jul 28;7:65. doi: 10.1186/1479-5876-7-65 (PMC2732596; doi:10.1186/1479-5876-7-65)

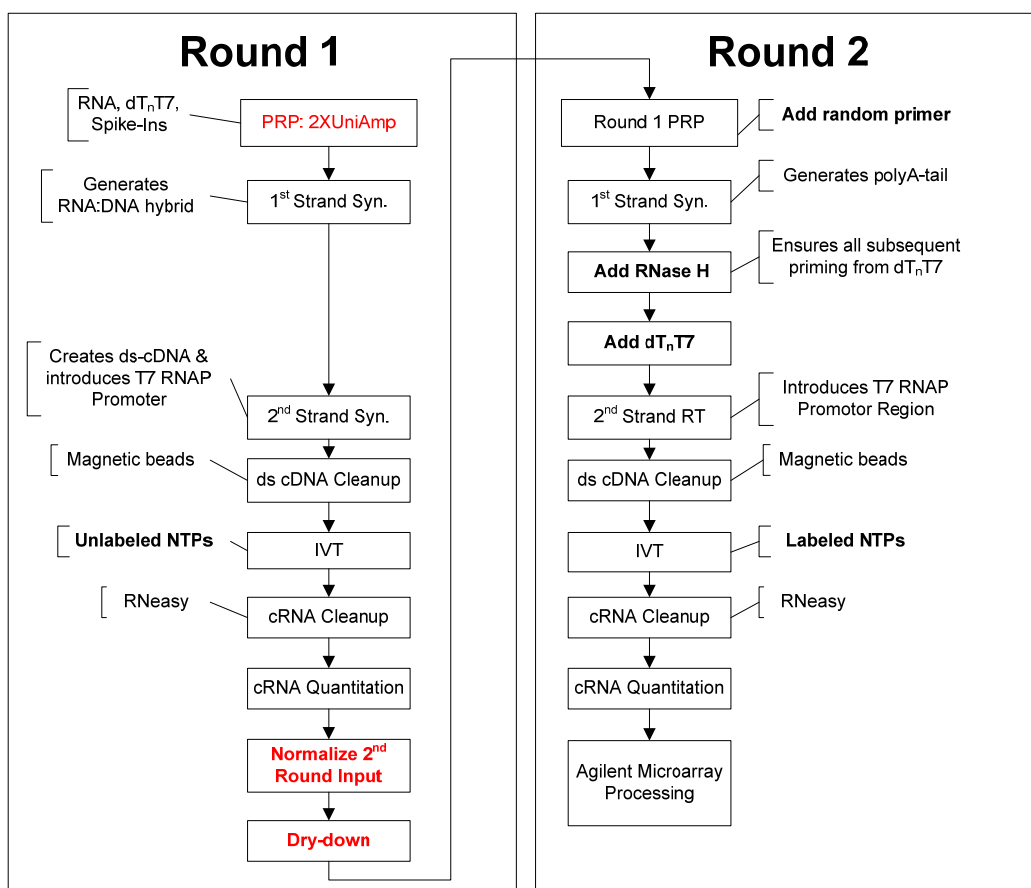

Supplement: Additional file 1 — Two-round amplification workflow. Diagram of the Two-round amplification work flow used for amplification of total RNA [file 1479-5876-7-65-S1.pdf]

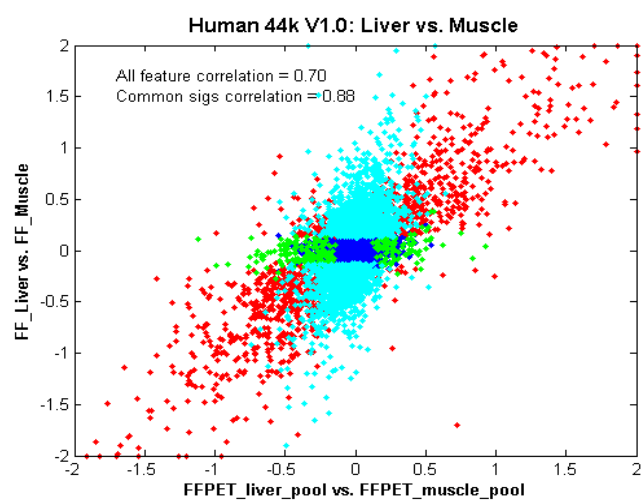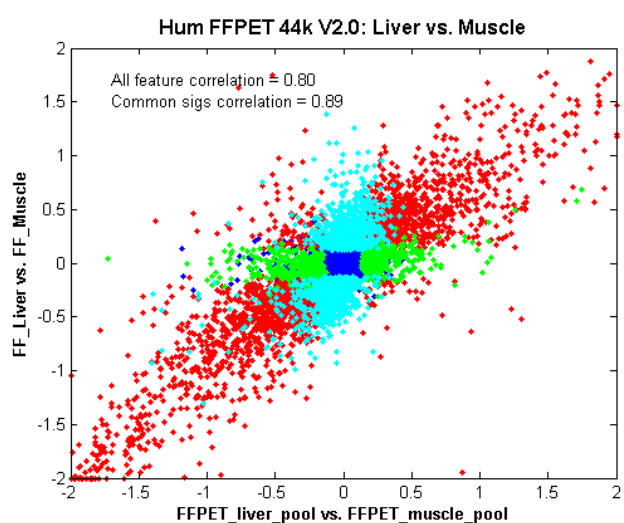

Supplement: Additional file 2 — FF to FFPET correlation. The correlation between the FF and FFPET samples is increased significantly on the HumFFPET 44 k array 2.0 when compared to the Human 44 k v1.1 array. [file 1479-5876-7-65-S2.pdf]
